# Global research frontiers and thematic trends in opioid-free anesthesia over the past 20 years: a bibliometric analysis

**DOI:** 10.3389/fphar.2025.1562765

**Published:** 2025-04-02

**Authors:** Junchen He, Rong Huang, Yingzhu Liu, Ying Chen, Min Zhong

**Affiliations:** ^1^ The Second Clinical Medical College, Guangzhou University of Chinese Medicine, Guangzhou, China; ^2^ Department of Anesthesiology, Guangdong Provincial Hospital of Traditional Chinese Medicine, The Second Affiliated Hospital of Guangzhou University of Chinese Medicine, Guangzhou, China

**Keywords:** opioid-free anesthesia, bibliometric analysis, citespace, multimodal analgesia, postoperative nausea and vomiting, plane block

## Abstract

**Objective:**

Opioids have constituted an essential element of general anesthesia for a considerable length of time. However, with the increase in opioid misuse and associated postoperative adverse effects, studies related to opioid-free anesthesia (OFA) have emerged, which pose a challenge in identifying key research directions. Accordingly, the objective of this study was to provide a review of the relevant literature in the field of OFA over the past 2 decades, with the goal of identifying the prevailing trends and research Frontiers.

**Methods:**

A systematic review of the publications on OFA was conducted using the Web of Science Core Collection database, with the objective of identifying relevant publications between the years 2005 and 2024. The bibliometric analysis was conducted using CiteSpace (version 6.1. R6), VOSviewer (version 1.6.19), and R (version4.4.2).

**Results:**

In conclusion, 477 publications were included in this study. The number of annual publications in this field has exhibited a steady increase over the past 2 decades. The United States and its institutions were found to be the most central. Forget, Patrice, and BELOEIL H were identified as the most prolific and highly cited authors, respectively. The journal with the highest number of publications was *BMC Anesthesiology*. The most frequently cited journal was *Anesthesia and Analgesia*, followed by *Anesthesiology*. In addition, keyword burst, keywords co-occurrence, and analysis of cited references indicate that recent studies have focused on: opioid consumption, pain, and postoperative nausea and vomiting (PONV). Meanwhile, analysis of keyword clusters and keywords timeline view showed that the main research frontiers are sevoflurane anesthesia, plane block, multimodal anesthesia, opioid-sparing anesthesia.

**Conclusion:**

Our results show that the current trends and directions of research focus on opioid consumption, pain, and PONV. Frontiers for future research are expected to include research areas related to sevoflurane anesthesia, plane block, multimodal anesthesia, opioid-sparing anesthesia.

## 1 Introduction

As μ-opioid receptor agonists, opioids have always been a ubiquitous component of general anesthesia regimens due to their potent analgesic properties (Weir, 2024). During surgical procedures, opioids primarily serve to manage pain, mitigate surgical stress responses, and reduce the requirement for volatile anesthetics under the operative circumstance. However, evidence from extensive opioid researches are paying more attention on their associated adverse effects, including respiratory depression, constipation, postoperative nausea and vomiting (PONV) (Huang et al., 2024), and opioid-induced hyperalgesia (OIH) (Chen L. et al., 2023). These complications may delay recovery and potentially contribute to significant morbidity and mortality.

In response to these safety concerns, opioid-free anesthesia (OFA) has emerged as a pivotal advancement in contemporary clinical practice (Mulier and Dekock, 2017). Although no universally accepted definition exists across clinical and academic communities, OFA fundamentally represents a multimodal strategy that maximizes non-opioid pharmacological agents and techniques, such as regional anesthesia, nerve blocks, and adjuvant analgesics, to achieve comprehensive perioperative anesthesia and analgesia while maintaining opioid accessibility for contingency use. This approach synergistically combines non-opioid medications targeting distinct pathways (Clanet et al., 2024), including dexmedetomidine, dexamethasone, lidocaine, magnesium, NSAIDs, and ketamine (Chen et al., 2024), to block nociceptive signal transmission.

Recent clinical trials have demonstrated that OFA improves postoperative outcomes in certain surgical populations. For instance, in patients undergoing thoracoscopic lung resection, OFA has been shown to reduce the incidence of PONV(Feng et al., 2024), while also significantly alleviating postoperative pain and decreasing opioid consumption (Toleska and Dimitrovski, 2019; Ibrahim et al., 2022; Zhou et al., 2023). Nevertheless, the clinical benefits of OFA remain controversial. A randomized controlled trial (RCT) involving patients undergoing ambulatory total hip arthroplasty revealed that intraoperative OFA failed to reduce opioid consumption within the first 24 h postoperatively and showed no significant differences in recovery outcomes or adverse event rates compared to opioid-based approaches (Chassery et al., 2024). Similar contradictory findings have been observed in clinical studies of gynecologic laparoscopic surgery (Massoth et al., 2021). This heterogeneity in therapeutic efficacy underscores the complexity of evaluating OFA’s clinical applicability.

Notably, despite 2 decades of global research efforts on OFA, the complexity of existing literature impedes systematic comprehension of its scientific evolution. Traditional narrative reviews remain constrained by subjective biases and fragmented analysis, whereas bibliometrics, an established quantitative discipline, enables objective trend mapping through data-driven modeling. This study therefore conducts a bibliometric analysis of OFA literature spanning the past 20 years, constructing visual models to delineate current research landscapes and emerging directions. The findings aim to provide structured references for optimizing OFA implementation and guiding future investigations.

## 2 Methods

### 2.1 Data sources and search strategies

In this study, the Web of Science Core Collection (WoSCC) was used to retrieve articles related to the topic (Peng et al., 2022). Data extraction was conducted on 8 December 2024. The search strategy was as follows: ALL = (opioid free anaesthesia) OR ALL = (opioid free anesthesia) OR ALL = (opioid-free anaesthesia) OR ALL = (opioid-free anesthesia) OR ALL = (opioid free general anaesthesia) OR ALL = (opioid free general anesthesia) OR ALL = (opioid-free general anaesthesia) OR ALL = (opioid-free general anesthesia) OR ALL = (anaesthesia without opioid) OR ALL = (anesthesia without opioid). The search period was limited to 1 January 2005 through 8 December 2024.

### 2.2 Inclusion and exclusion criteria

The inclusion criteria for the study were (1) original articles or review articles; (2) written in English; The exclusion criteria were (1) the research topic was not related to OFA; (2) the article types were abstract, book chapter, editorial, letter, meeting, retracted publication, proceeding paper,and the others; (3) articles with an unavailable abstract and a digital object identifier (DOI) number; (4) articles for which the full text were not available. The literature search, data extraction, and downloads were completed on 8 December 2024. The two groups of reviewers searched and collected data independently, with disagreement referred to arbitration by a third researcher. A total of 477 articles were included.

### 2.3 Statistical analysis and visualization

CiteSpace (version 6.1.R6), VOSviewer (version 1.6.19), and R (version 4.4.2) “the bibliometrix package” were used to analyze and visualize the data.

Professor Chaomei Chen developed this CiteSpace software, which runs in Java environment. The software allows for the visual presentation of literature-related information, such as country, institution, author, and keywords (Chen and Song, 2019). In the visual maps we generated using CiteSpace, each node corresponds to a country, institution, journal, author, or keyword. The size of the node indicates the frequency of occurrence or citation, and the color indicates the year. The centrality of each node is indicated by the outermost purple ring of the node, the thickness of which represents the degree of centrality. The cluster analysis used modularity (Q-value) and average contour value (S-value) to evaluate the overall structural characteristics of the network, and the clustering quality of the cluster map was considered satisfactory when Q > 0.3 and S > 0.5.

VOSviewer is software for building and viewing bibliometric maps. It can be used to construct author or journal maps, or to construct keyword co-occurrence networks (Zhu et al., 2022).

The “bibliometrix package” is an R-based bibliometric analysis tool that provides various data presentation functions and visualization tools for tracking the time of research topics and for conducting journal analysis (Aria and Cuccurullo, 2017).

## 3 Results

**FIGURE 1:**
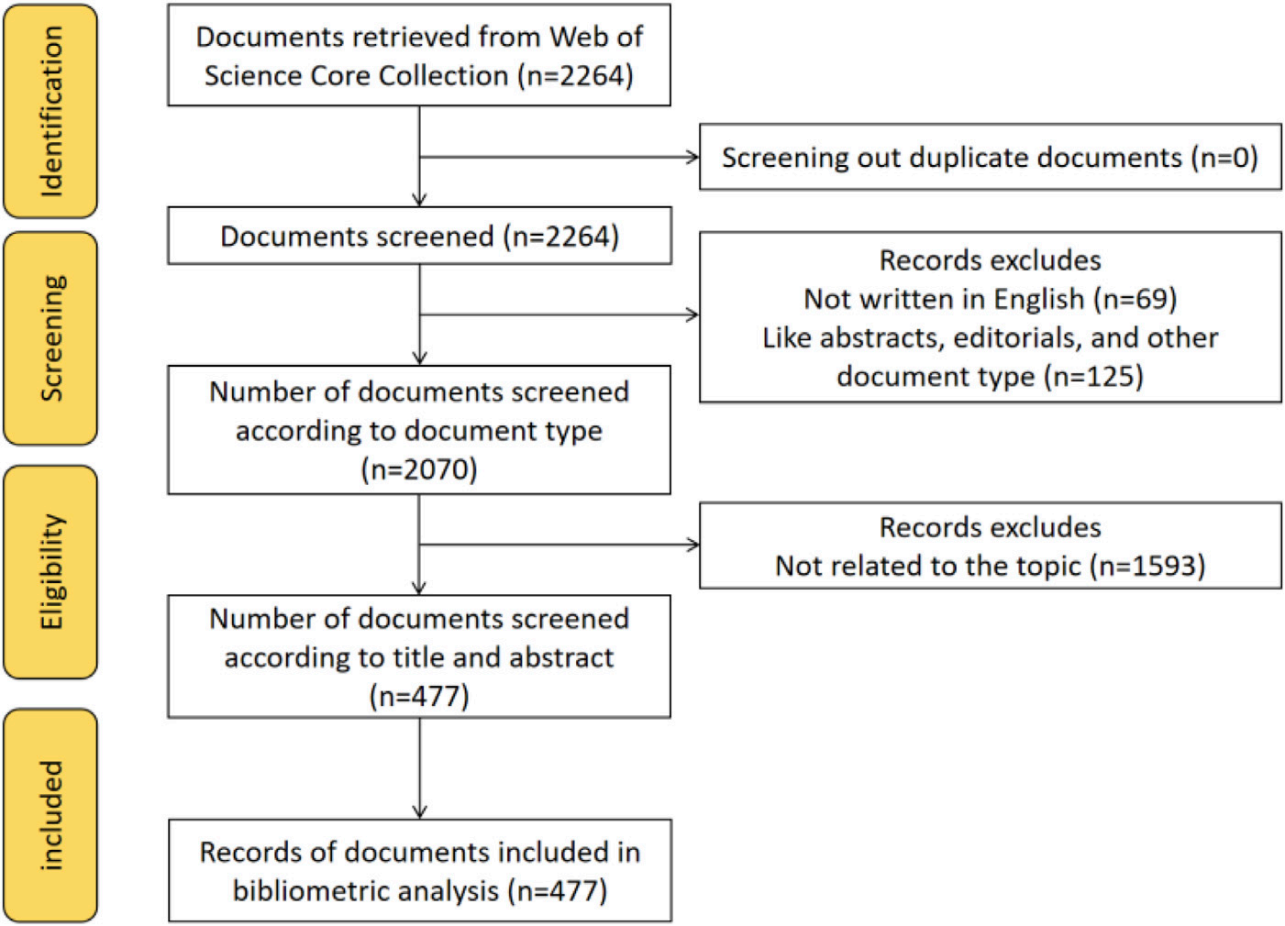
PRISMA flowchart of the study.

Between 1 January 2005, and 8 December 2024, 2,264 OFA-related publications were published. Firstly, we excluded some specific types of publications, such as meetings, letters and the others, as well as some non-English publications. In the end, 2,070 publications remained. Subsequently, both groups of researchers carefully reviewed the titles and abstracts of 2070 publications (reviewing the full text, if necessary), with a third person judging if the two disagreed, to make sure that the included literature all met the inclusion criteria, to ensure the accuracy of the data, and to exclude articles whose studies were not relevant to OFA. Eventually, a total of 477 publications met the inclusion criteria (Figure 1) and were included in the analysis, including 395 articles and 82 reviews.

### 3.1 Bibliometric analysis of annual global publication outputs

**FIGURE 2:**
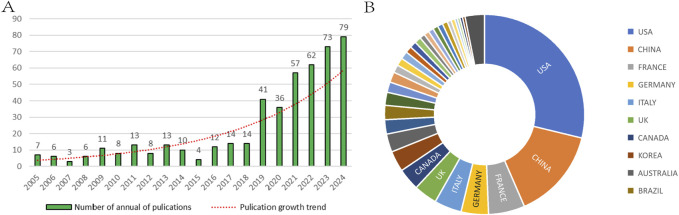
Bibliometric analysis of publication outputs. **(A)** The number of annual publications. **(B)** The distribution of publications by countries/regions; Each module represents a different country/region.

Annual publication volume is a reflection of research output and the level of activity in specific research areas. This bibliometric study included and analyzed 477 papers that met the inclusion criteria between 2005 and 2024. Figure 2A shows the change in annual publications on research related to the OFA research area over the 20 years since 2005. As can be seen from the figure, there has been a gradual increase in the number of publications related to the topic since 2005. From 2005 to 2024, the number of articles increased almost tenfold (7 and 79, respectively). From 2005 to 2018, the increase in the number of publications is lower, even decreasing to 4 articles in 2015, suggesting that there may have been a difficult period of exploration in the development of OFA during this period. However, in 2019, OFA experienced rapid growth with a sudden increase to 41 publications. This was followed by a steady increase to 79 publications in 2024, although there was a slight decrease in 2020. Overall, these findings highlight the growing interest in OFA. Our results showed that studies on OFA were conducted in 50 different countries/regions, and the United States had the largest number of publications (n = 164), accounting for 34.4% of the total. China (n = 71, 14.9%) was the next most common country (Figure 2B).

### 3.2 Analysis of countries/regions distribution

**FIGURE 3:**
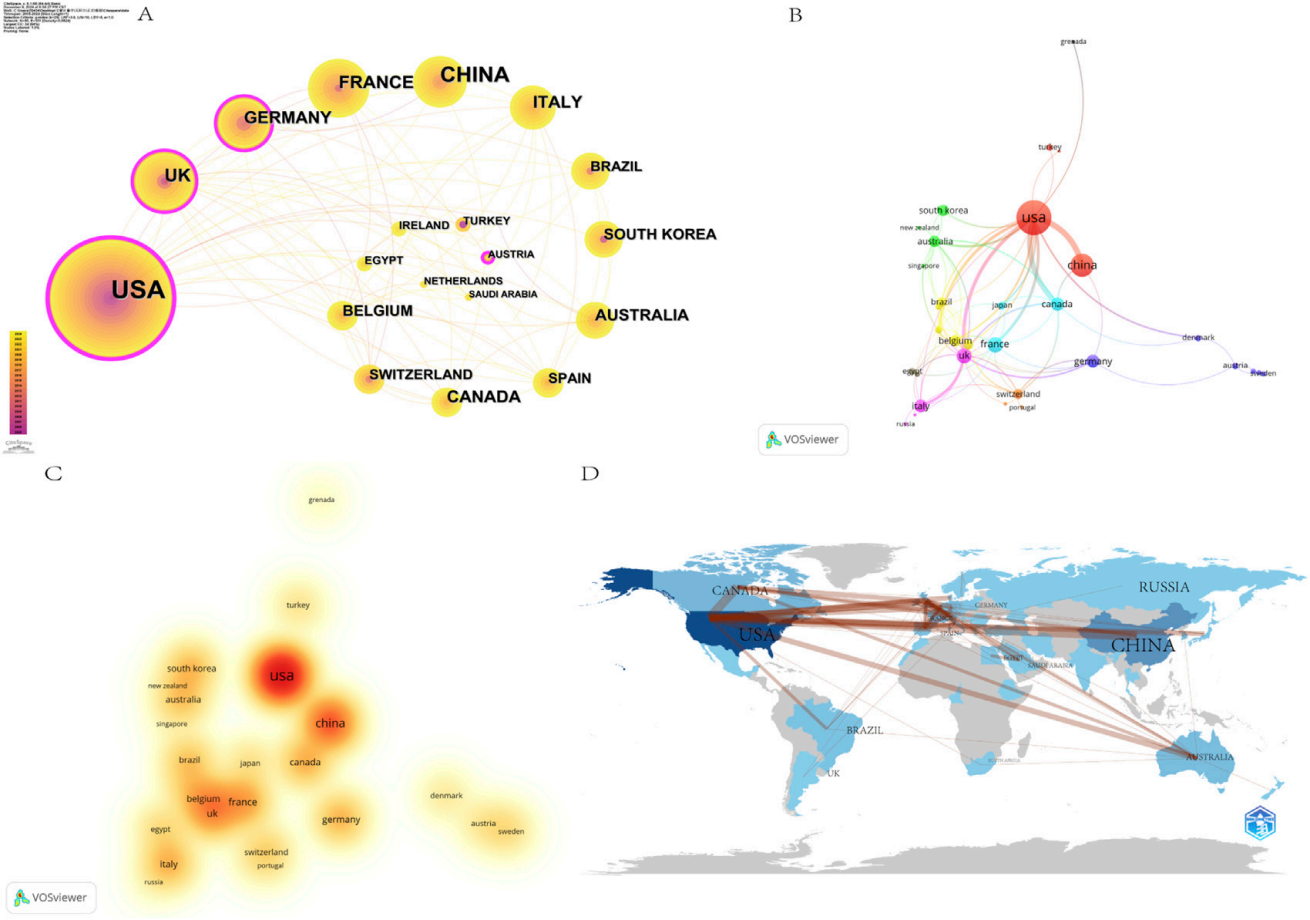
Cooperation between countries/regions on OFA. **(A)** The cooperation networks between different countries/regions; **(B)** The network visualization map of countries/regions based on VOSviewer; **(C)** The density visualization map of countries/regions based on VOSviewer; **(D)** The visualization map of the published national cooperative geographic network.

For China, publications from Taiwan were included, and for the United Kingdom, publications from England, Wales, Scotland, and Northern Ireland were included. The 477 papers analyzed in this study were completed by 525 authors from 382 institutions in 50 countries. The five countries with the largest number of publications were the United States (n = 164), China (n = 71), France (n = 33), the United Kingdom (n = 27) and Italy (n = 22) (Table 1). The calculation of centrality allows further assessment of the level of significance and influence of each node in the visualization network. The United States leads the other countries in this research topic with 164 publications. Furthermore, the United States also stands out with a centrality of 0.29, which implies that there is a close and frequent collaboration between US and other countries (Figures 3A, B), which also establishes the United States as the center of this field of research. This also highlights the tendency of the United States researchers to engage in international collaborations for research purposes (Figures 3A, C, D). For a geographical perspective on collaboration, Figure 3D presents a graph with darker shades indicating higher publication counts and thicker lines signifying stronger collaborative ties. China is the second-leading contributor, with a significantly higher number of publications than other countries (n = 71). However, its centrality is comparatively lower (0.01). This phenomenon may be attributed to China’s comparatively delayed initiation of research in OFA-related domains, compounded by language barriers that hinder effective communication with other nations. These factors potentially contribute to the reduced collaborative nature of China’s research in this field. Notwithstanding these circumstances, China’s publication output is nevertheless second in terms of quantity, underscoring the nation’s noteworthy endeavors and considerable potential in the realm of international research collaboration.

**TABLE 1:** The top 10 countries/regions and institutions involved in OFA.

Rank	Countries/Regions	Count	Centrality	Institutions	Count	Centrality	Country
1	United States	164	0.29	Duke Univ	15	0.04	United States
2	China	71	0.01	Stanford Univ	11	0.01	United States
3	France	33	0.02	Cleveland Clin	10	0.09	United States
4	United Kingdom	27	0.09	Univ Toronto	10	0.03	Canada
5	Italy	22	0.01	Univ Aberdeen	9	0.05	United Kingdom
6	Canada	21	0.02	Fudan Univ	8	0.04	China
7	Germany	19	0.04	Harvard Med Sch	7	0.07	United States
8	Belgium	17	0.02	Univ Calif San Francisco	7	0.01	United States
9	South Korea	17	0.00	Massachusetts Gen Hosp	6	0.03	United States
10	Australia	16	0.02	Capital Med Univ	6	0	China

### 3.3 Analysis of institutions

**FIGURE 4:**
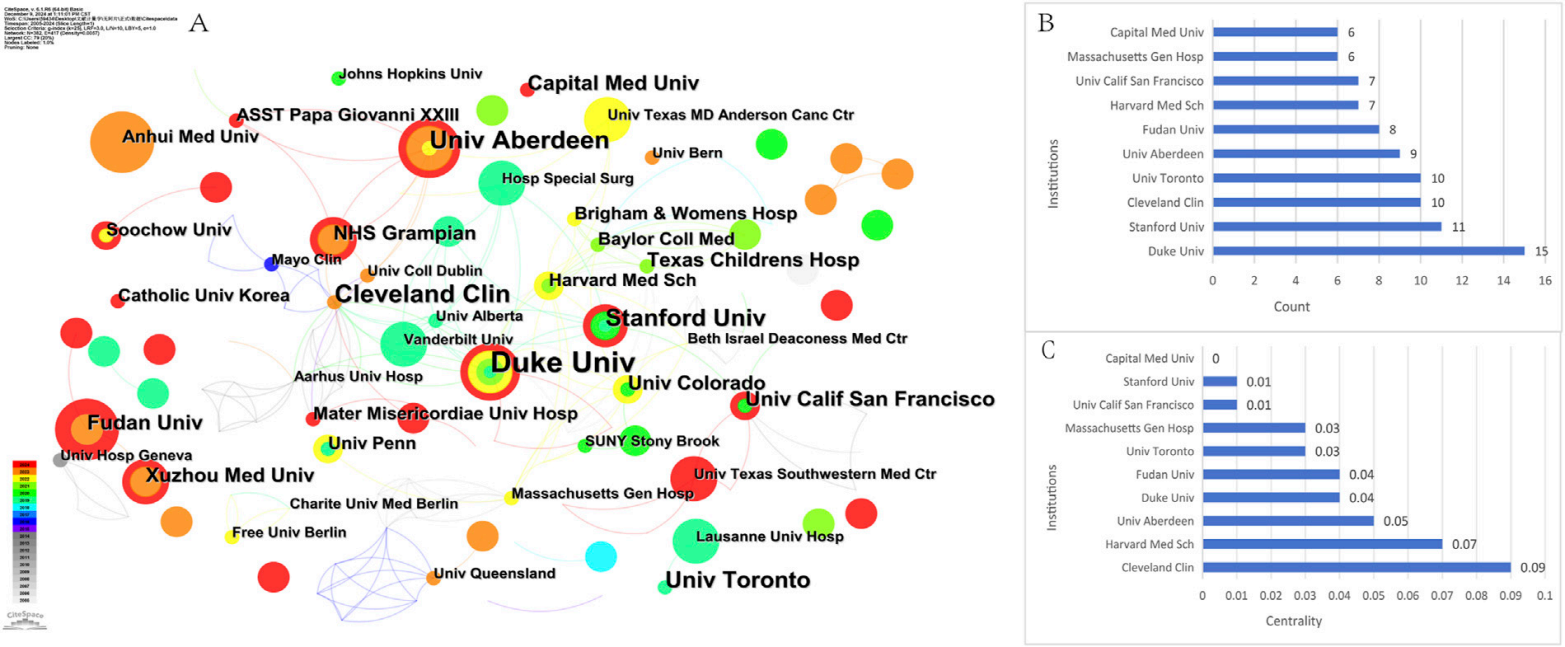
Cooperation between institutions on OFA. **(A)** The cooperation networks between different institutions; **(B)** The top 10 institutions in count; **(C)** The top 10 institutions in centrality.

To understand and assess the academic influence and contribution of each research institution in the field, the top ten institutions with most publications were examined (Table 1). The institutions ranked in descending order by the number of publications are Duke University (the United States, n = 15), Stanford University (the United States, n = 11), the Cleveland Clinic (the United States, n = 10), University of Toronto (Canada, n = 10), and University of Aberdeen (United Kingdom, n = 9) (Figures 4A, B). Regarding centrality, Cleveland Clinic had the highest centrality of overseas institutions (centrality = 0.09), which positioned it as a core institution in the overseas research field (Figures 4A, C). The second highest ranked institution was Harvard Medical School (centrality = 0.07). This may indicate that these institutions have a strong research focus or expertise in the area of OFA research.

### 3.4 Analysis of authors and cited authors

**FIGURE 5:**
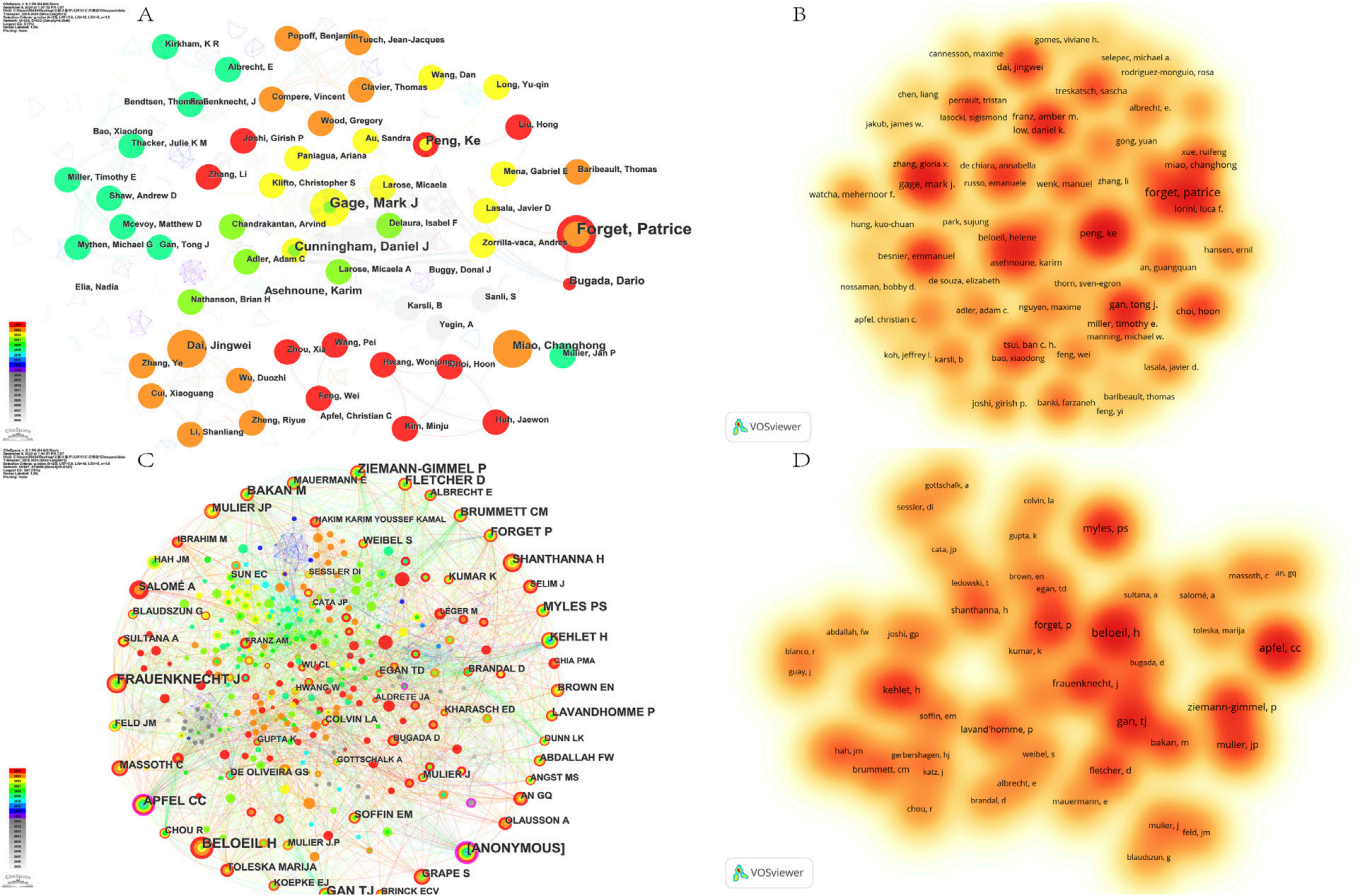
Authors analysis on OFA. **(A)** The cooperation networks between different authors; **(B)** The density visualization map of authors based on VOSviewer; **(C)** The cooperation networks between different cited authors; **(D)** The density visualization map of cited authors based on VOSviewer.

The analysis of Figure 5A shows a large number of author nodes, suggesting a close network of collaborative relationships among authors in the domain. In addition, the observed high connectivity strength indicates close collaboration between the authors. Price proposed that half of the papers in the same domain are produced by highly productive authors (Price, 1963). According to Price’s law, the minimum publication volume for core authors is N = 0.749×√n_max_. n_max_ denotes the volume of papers by the most productive authors. N was calculated to be 1.98. Authors with more than two publications were considered core authors in the field by rounding up. Finally, we identified 65 core authors with a total of 146 publications, accounting for 30.6% of the total number of publications by all authors. The top three authors were those with more than 2 publications, which were rounded up to be considered core authors in the field. The top three authors are Forget, Patrice (n = 7), Gage, Mark J (n = 5), and Peng, Ke (n = 4). All of the above authors have a centrality below 0.01 (denoted as 0.00), suggesting that there is a certain gap in research in this area, with no notable authors (Table 2) (Figure 5B). However, among these authors, Asehnoune, Karim, and Joshi, Girish P have a relatively high H-index of 51 and 50 respectively, suggesting that the importance of the need for further research as well as a huge scope for research. The most cited of the co-cited authors is BELOEIL H (n = 81), who has shown great interest in the field of OFA and can be considered an expert in this area (Figures 5C, D) (Table 2).

**TABLE 2:** The top 10 authors and cited authors in OFA.

Rank	Author	Count	Centrality	H-index	Cited author	Count	Centrality
1	Forget, Patrice	7	0.00	35	BELOEIL H	81	0.01
2	Gage, Mark J	5	0.00	13	[ANONYMOUS]	67	0.24
3	Peng, Ke	4	0.00	14	FRAUENKNECHT J	65	0.03
4	Cunningham, Daniel J	4	0.00	13	ZIEMANN-GIMMEL P	60	0.04
5	Asehnoune, Karim	3	0.00	51	GAN TJ	56	0.09
6	Miao, Changhong	3	0.00	21	APFEL CC	53	0.16
7	Bugada, Dario	3	0.00	17	MYLES PS	52	0.02
8	Dai, Jingwei	3	0.00	18	BAKAN M	51	0.07
9	Joshi, Girish P	2	0.00	50	FLETCHER D	47	0.05
10	Beloeil, Helene	2	0.00	36	FORGET P	45	0.07

### 3.5 Analysis of journals

**FIGURE 6:**
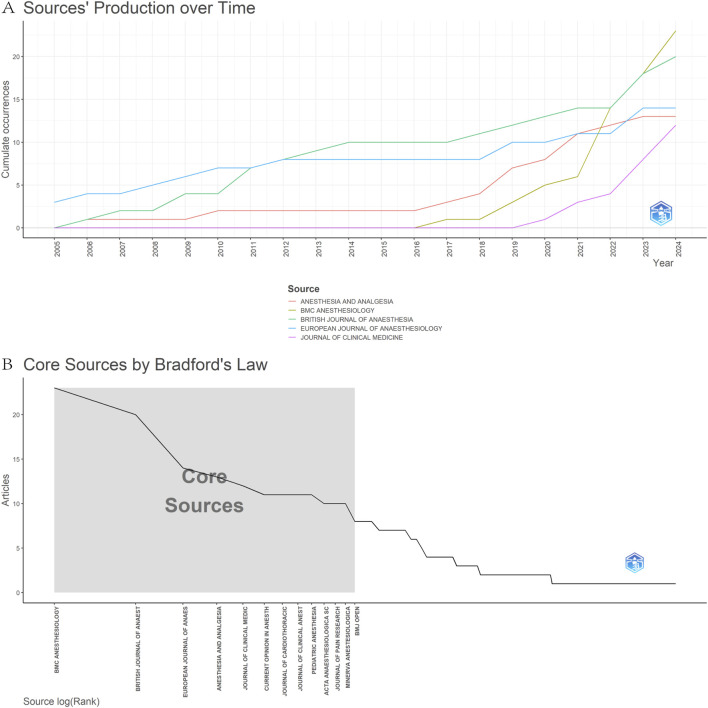
Journals analysis on OFA. **(A)** Journals output trends within the top 5 from 2005 to 2024; **(B)** Core journals by Bradford’s law.

**FIGURE 7:**
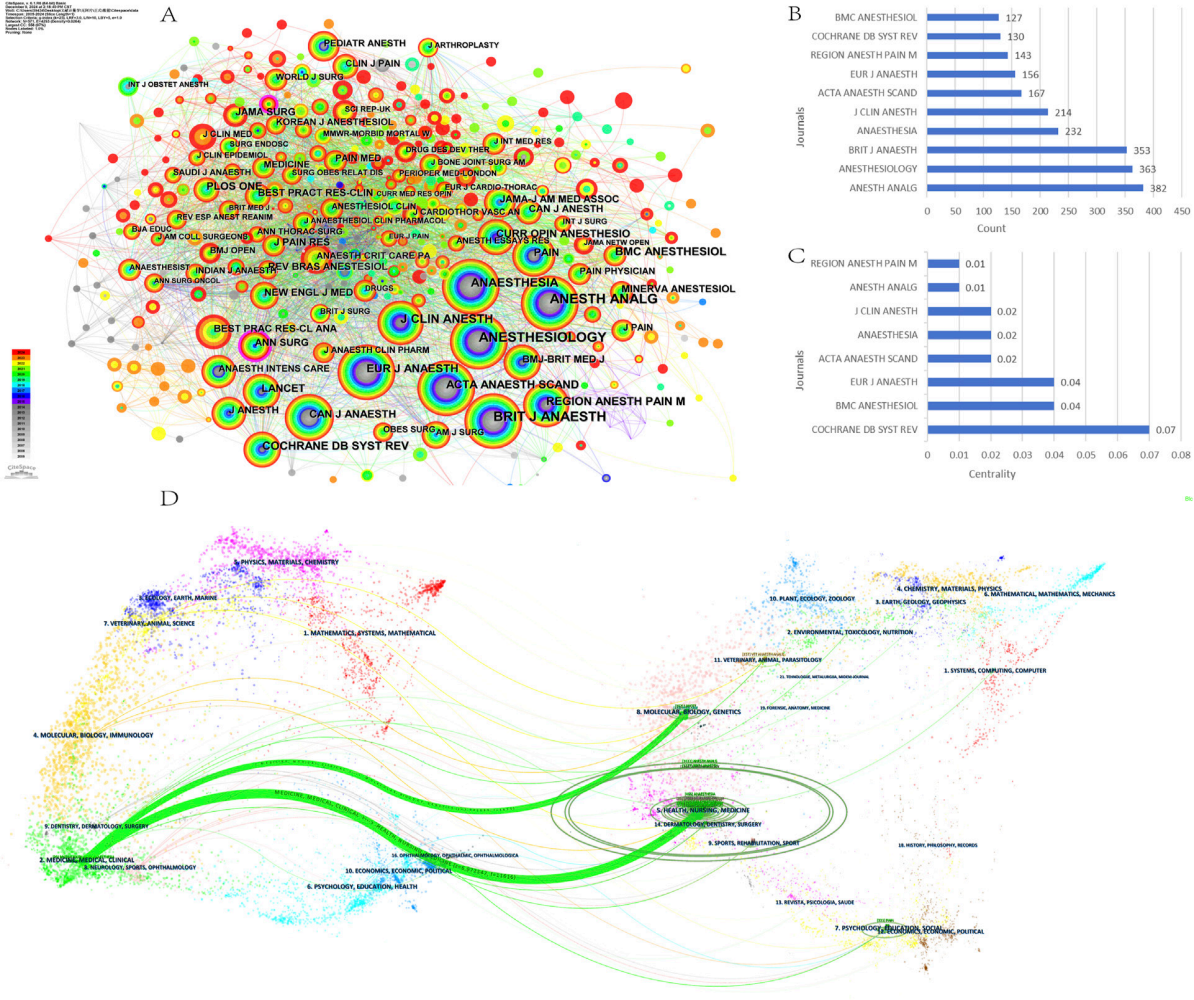
Cited journals analysis on OFA. **(A)** The network map of cited journals; **(B)** The top 10 cited journals in count; **(C)** The top 10 cited journals in centrality; **(D)** The dual-map overlay of citing of citation relationship of articles, with citing journal on the left, and the cited journal on the right. The colored path represented the citation relationship.

Figure 6A presents the top 5 journals ranked by the number of publications, with a significant upward trend from 2005 to the present. According to Bradford’s law, 201 journals were divided into zones 1–3, with zone 1 containing 13 core journals such as *BMC Anesthesiology* and *British Journal OF Anaesthesia* (Figure 6B) (Table 3). The co-citation network visualization of the journals was generated by CiteSpace software (Figure 7A). The results showed that the most cited journal was *Anesthesia & Analgesia* (n = 382), followed by *Anesthesiology* (n = 363) and *British Journal of Anaesthesia* (n = 353) (Figure 7B) (Table 4). Among these journals, *the Cochrane Database of Systematic Reviews* had the highest centrality (centrality = 0.07) (Figure 7C). The journals mentioned above are some of the most prominent journals in the field of anesthesiology, which have contributed to the development of anesthesiology and are known for their wide impact and authority.

**TABLE 3:** According to Bradford’s Law, the 201 Journals in OFA were Classified into Zones 1–3.

Zone	Number of journals	Number of publications	Percentage
1	13	164	34.4%
2	44	157	32.9%
3	144	156	32.7%
Total	201	477	100%

**TABLE 4:** The top 10 cited journals in OFA.

Rank	Journal	Count	Centrality	If (2024)	JCR (2024)
1	Anesthesia and Analgesia	382	0.01	4.6	Q1
2	Anesthesiology	363	0	9.1	Q1
3	British Journal of Anaesthesia	353	0	9.1	Q1
4	Anaesthesia	232	0.02	7.5	Q1
5	Journal of Clinical Anesthesia	214	0.02	5.0	Q1
6	Acta Anaesthesiologica Scandinavica	167	0.02	1.9	Q2
7	European Journal of Anaesthesiology	156	0.04	4.2	Q1
8	Regional Anesthesia and Pain Medicine	143	0.01	5.1	Q1
9	Cochrane Database of Systematic Reviews	130	0.07	8.8	Q1
10	BMC Anesthesiology	127	0.04	2.3	Q2

The dual-map overlay of journals shows the overall contribution of the science. In the dual-map overlay, each node represents a journal, with citing journals on the left, the journals we included in the study for publication, and cited journals on the right, the journals cited in the literature included in the study. The lines connecting the left and right sides of the map correspond to the citation relationship between the journals, and the thicker the connecting line indicates the strength of the knowledge flow relationship between the journals indicating the rush crowd (Chen et al., 2022). In the dual-map overlay of journals in this study (Figure 7D), there are two outward citation paths in the citing journals section on the left side of the map, which include surgery, medicine, and neurology. At the same time, molecular, nursing, and biology served as the main sources, with the highest citation rates in the disciplines of health, medicine, and nursing (Z value = 6.972, F value = 11,616).

### 3.6 Analysis of cited references

**FIGURE 8:**
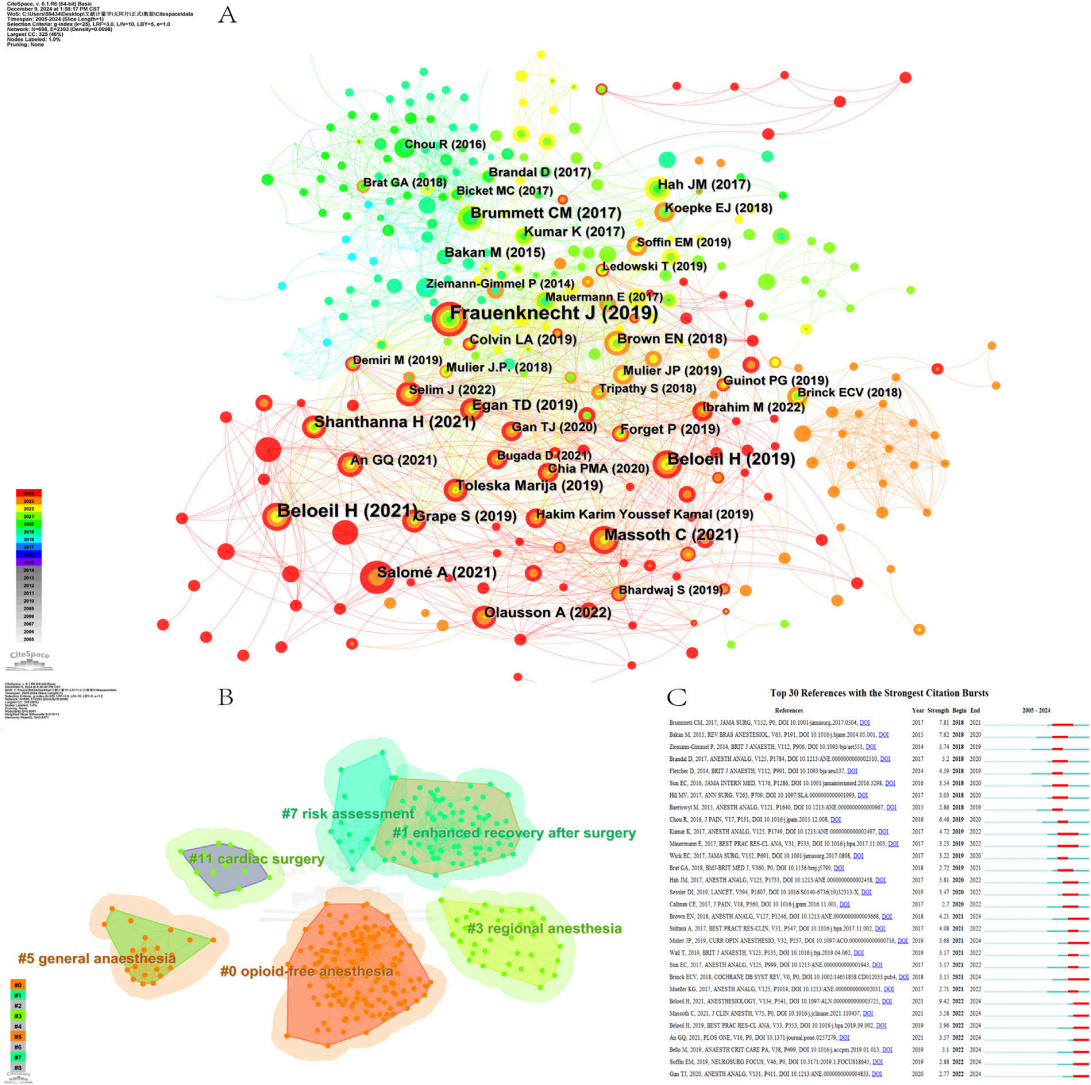
Cited references analysis on OFA. **(A)** The network map of cited references; **(B)** The clustering of cited references; **(C)** The top 30 cited references with the strongest citation bursts.

In this study, we used CiteSpace to identify cited references. The analysis of cited references is essential and has a crucial reference value in determining the research outlook and emerging trends within the field of study. A total of 698 references were detected, of which 65 studies had a centrality greater than 0.01. Table 5 shows the top 10 most cited references. These studies play a key role in the development of the field, playing the role of classic basic research at different times, and have largely promoted the development of the field. The article “Analgesic impact of intra-operative opioids vs. opioid-free anaesthesia: a systematic review and meta-analysis,” published by Frauenknecht J, was the most cited article, with 64 citations (Figure 8A). Cluster analysis of the cited references revealed 6 major clusters (Q-value = 0.8091, S-value = 0.9111): #0 opioid-free anesthesia, #1 enhanced recovery after surgery, #3 regional anesthesia, #5 general anesthesia, #7 risk assessment, and #11 cardiac surgery (Figure 8B). The citation frequency of the top ten most cited papers together was more than 20, with a particular emphasis on cluster #0 opioid-free anesthesia (n = 9) and cluster #1 enhanced recovery after surgery (n = 1).

**TABLE 5:** The top 10 cited references in OFA.

Rank				References			Count			Centrality			Publication Year			
1				Frauenknecht et al. (2019)			64			0.04			2019			
2				Beloeil et al. (2021)			57			0.01			2021			
3				Beloeil (2019)			34			0.02			2019			
4				Shanthanna et al. (2021)			29			0.02			2021			
5				Massoth et al. (2021)			28			0.01			2021			
6				Salomé et al. (2021)			27			0			2021			
7				Brummett et al. (2017)			24			0.03			2017			
8				Grape et al. (2019)			22			0.02			2019			
9				Toleska and Dimitrovski (2019)			21			0.01			2019			
10				Egan (2019)			21			0.05			2019			

The burst strength of references is often related to the direction of future research, and references with high burst strength tend to cover a broader range of scientific discoveries. The two references with the highest burst strength were “Balanced Opioid-free Anesthesia with Dexmedetomidine versus Balanced Anesthesia with Remifentanil for Major or Intermediate Noncardiac Surgery” (strength = 9.42) and “New Persistent Opioid Use After Minor and Major Surgical Procedures in US Adults” (strength = 7.81) (Figure 8C).

There are currently 7 pieces of literature that are still in a state of burst, which are: “Balanced Opioid-free Anesthesia with Dexmedetomidine versus Balanced Anesthesia with Remifentanil for Major or Intermediate Noncardiac Surgery”, “Impact of opioid-free anaesthesia on postoperative nausea, vomiting and pain after gynaecological laparoscopy - A randomised controlled trial”, “Opioid-free anesthesia”, “Opioid-free anesthesia compared to opioid anesthesia for lung cancer patients undergoing video-assisted thoracoscopic surgery: A randomized controlled study”, “Effect of opioid-free anaesthesia on postoperative epidural ropivacaine requirement after thoracic surgery: A retrospective unmatched case-control study”, “Opioid-free anesthesia within an enhanced recovery after surgery pathway for minimally invasive lumbar spine surgery: a retrospective matched cohort study”, “Fourth Consensus Guidelines for the Management of Postoperative Nausea and Vomiting.”Among the literature that is currently still in an burst state, more than two focus on the theme of PONV, indicating that PONV is a significant research hotspot.

### 3.7 Analysis of subject categories and research themes

According to the WOS categories, the 477 articles on OFA covered 31 topics, and Table 6 shows the top 10 topics. Among these, the top research topic was “Anesthesiology” (n = 203), followed by “Medicine, General & Internal” (n = 60), “Clinical Neurology” (n = 24), and “Critical Care Medicine” (n = 22).

**TABLE 6:** Top 10 productive WOS categories in OFA, ranked by the number of publications.

Rank	Web of science categories	Record count
1	Anesthesiology	203
2	Medicine, General & Internal	60
3	Clinical Neurology	24
4	Critical Care Medicine	22
5	Surgery	18
6	Cardiac & Cardiovascular Systems	14
7	Pediatrics	12
8	Respiratory System	12
9	Gastroenterology & Hepatology	11
10	Peripheral Vascular Disease	11

**FIGURE 9:**
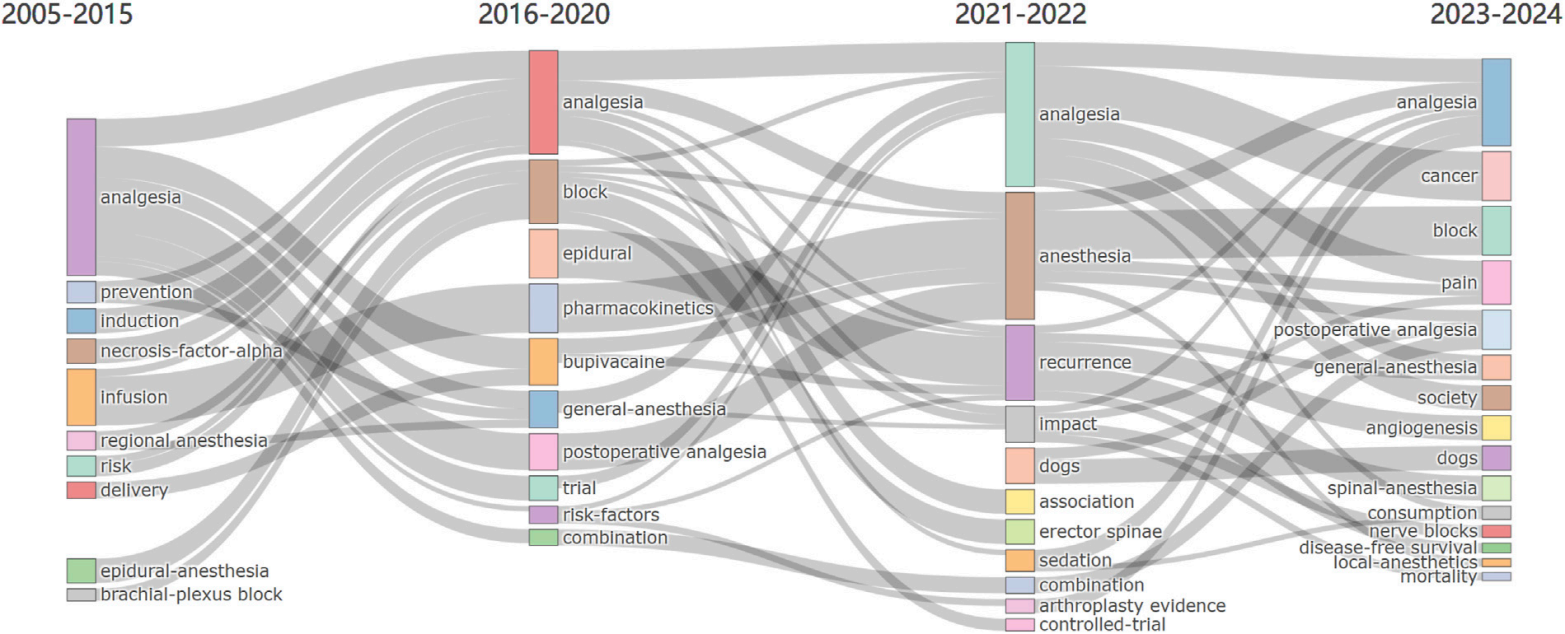
R bibliometrics-thematic evolution tool traces the progression of research themes in the realm of OFA, spanning from 2005 to 2024.

To more visually demonstrate the evolution of research themes over the past 20 years, we created a map of themes over time (Figure 9). Over the past 20 years, there have been some changes in research themes. From 2005 to 2014, research related to OFA primarily focused on 10 main themes, which included “analgesia,” “regional anesthesia,” and “epidural anesthesia.” However, over time, the research topics became more diverse. Between 2016 and 2024, the main themes included “analgesia,” “anesthesia,” “general anesthesia,” “block,” “pain,” “postoperative analgesia,” and “consumption”. Research on pain in anesthesia remains a key focus, indicating that there are still gaps in the study of pain management in this field. Future research is needed to further explore this area, with the aim of improving perioperative pain management measures and enhancing patient safety, satisfaction, and comfort.

### 3.8 Analysis of keywords

#### 3.8.1 Keywords Co-Occurrence

**FIGURE 10:**
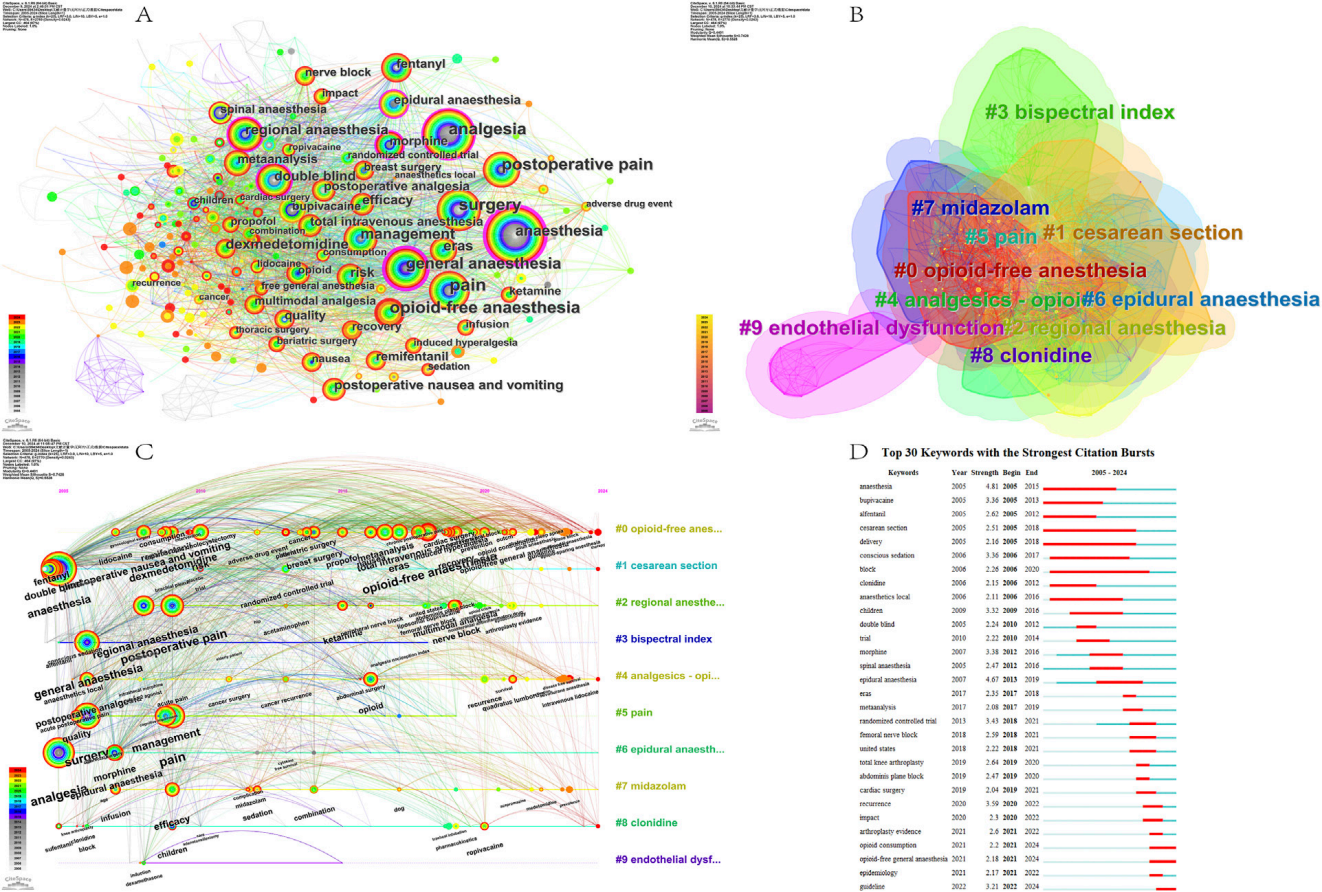
Keywords analysis on OFA. **(A)** The network map of the co-occurrence keywords; **(B)** Clusters of keywords; **(C)** Timeline view of keywords analysis; **(D)** The top 30 keywords with the strongest citation bursts.

The co-occurrence of keywords in the literature is analyzed, as well as the keywords that appear with high frequency in the literature are organized and classified, and the strength of association between individual keywords is observed. By using this method, we can better understand the internal structure and composition of OFA-related fields and explore the boundaries of these fields. Figure 10A shows the keyword co-occurrence map generated by CiteSpace. In the keyword co-occurrence map, the keywords with higher occurrence rate and higher centrality tend to be closer to the research frontiers or represent the research hotspots in the field. The keyword “pain” had the highest frequency of occurrence in this study (n = 123), followed by “analgesia” (n = 111). The centrality of “general anesthesia” and “anesthesia” was the highest at 0.25 and 0.23, respectively, indicating that the current research hotspots in OFA remain focused on the management of pain during the perioperative period under general anesthesia. The research frontiers are still focused on anesthesia-related areas.

#### 3.8.2 Keyword clusters

The cluster analysis of the keywords showed that 478 keywords could be classified into 10 main clusters (Q value = 0.4397, S value = 0.752): #0 opioid-free anesthesia, #1 cesarean section, #2 regional anesthesia, #3 bispectral index, #4 analgesics-opioid, #5 pain, #6 epidural anesthesia, #7 midazolam, #8 clonidine and #9 endothelial dysfunction (Figure 10B). The average year for cluster #0 was 2019, indicating that the research in question is in the midst of fast-moving research. In the cluster analysis, high-frequency keywords included “propofol”, “opioid-free anesthesia”, “analgesia”, “plane block”, “postoperative nausea”, “perioperative care”, “patient-reported outcome measures”, and “opioid-related adverse effects.” The use of propofol or various plane block techniques to reduce opioids and their associated adverse effects has become a prominent area of research in recent years.

#### 3.8.3 Keywords timeline view and analysis of research hotspot

To illustrate the regular situation of keywords over time, we created a keyword timeline view using CiteSpace. The keyword timeline view clearly visualizes the results and trends of the OFA research area since 2005 and also provides an effective visualization of its internal relationships. In the keyword timeline view, every node indicates a keyword, the size of the node correlates to the occurrence frequency of the keyword, and the change in node color corresponds to the trend over time.

In 2005, the keywords “analgesia”, “general anesthesia”, “fentanyl,” and “postoperative analgesia” were central. Not only did these keywords form the basis of this study, but they also maintained the focus of the research throughout the course of the study. From 2006 to 2022, research topics such as “postoperative nausea and vomiting,” “remifentanil,” “dexmedetomidine,” “total intravenous anesthesia,” “efficacy,” “eras,” and “opioid-free anesthesia” emerged. In 2023, emerging research areas included “intravenous lidocaine”, “sevoflurane anesthesia”, “opioid-sparing anesthesia”, “multimodal anesthesia”, and “plane block”. These results indicate that achieving OFA or opioid-sparing anesthesia (OSA) through multimodal anesthesia (MMA) strategies, such as the use of sevoflurane anesthesia, plane blocks, and other anesthetic techniques to reduce or completely avoid opioid use, has become a key focus for future research (Figure 10C).

#### 3.8.4 Keyword Bursts

CiteSpace can determine the current burst of keywords in the research field, and the burst map can effectively elaborate the time sequence of the burst of keywords, which is helpful in assessing the research direction and attention in a specific period. Over the past 20 years, “anesthesia” has been ranked number one with the highest burst strength (strength = 4.81). In particular, “opioid consumption”, “opioid-free general anesthesia” and “guideline” have received significant attention recently. They have been positioned as current research topics and trends (Figure 10D).

## 4 Discussion

The bibliometric analysis presents a visual representation of 477 publications on OFA. CiteSpace, Vosviewer, and R language were employed to elucidate the present state of research on OFA and to suggest potential future avenues for investigation.

### 4.1 Basic information

According to the findings of a comprehensive analysis of both qualitative and quantitative data conducted by CiteSpace, research activities related to OFA have exhibited an upward trend over the past 2 decades. It is worth noting that there was a significant increase in publications around 2019, possibly due to increased awareness of the key role of OFA among scientists. The United States (the first) and China (the second) are the two countries with the most research on OFA, and their scientific research investment is also very substantial. In this regard, the number of publications in the United States is ahead of China, more than twice that of China, and the centrality is the highest, which shows the central role of the United States in research and close international cooperation in this field. China’s strength may be somewhat weaker due to its late start in research in this area, as well as the lack of authoritative scholars in the field and fewer high-impact publications. In addition, there are extensive contacts and cooperation among countries and institutions, especially between China and the United States. Patrice Forget from the University of Aberdeen has made a great contribution to this field by publishing a number of studies (Bugada et al., 2022; Forget, 2022; Hublet et al., 2022). The most cited author is Helene Beloeil of Universite Rennes, who has done much research in the field of opioid use in the perioperative period and is considered an expert on the subject (Aldamluji et al., 2021; Anger et al., 2021; Beloeil et al., 2021). The most cited journals are *Anesthesia and Analgesia*, *Anesthesiology* and *British Journal of Anaesthesia*. These three journals hold significant importance in the domain of anesthesiology, having published numerous high-quality articles that have contributed substantially to the advancement of the field. The most frequently cited journal article is Analgesic impact of intra-operative opioids vs. opioid-free anaesthesia: a systematic review and meta-analysis by J Frauenknecht et al. (Frauenknecht et al., 2019), published in the journal *Anaesthesia*. A total of 23 RCTs were included into this meta-analysis, and the paper systematically examined the impact of intraoperative opioid analgesic utilization on postoperative pain and other adverse effects, which held significant reference value for anesthesiologists, as it provided guidance on the adjustment of intraoperative anesthetic medications.

### 4.2 Theme trends and hot topics

In this study, we aim to analyze OFA-related fields in order to provide a comprehensive summary analysis of research frontiers and hotspots. Within a paper, keywords, as a part of the paper throughout the text, are extremely important to the overall conceptualization and structural construction of the paper, and it can be said that the keywords determine the direction of the paper. Emerging research themes can be identified by analyzing the frequency of keywords and combining them with literature that was highly cited over a specific time period. These trends and research themes reflect areas that have been the subject of considerable academic attention and interest over a specific period of time. We hope that scholars interested in this area will be able to gain some convenience and reduce duplication of effort.

#### 4.2.1 Pain and opioid consumption

Through the analysis of Keyword Bursts and keyword co-occurrence, we have identified “Pain” and “Opioid Consumption” as two critical research themes. The most widely accepted definition of pain is provided by the International Association for the Study of Pain: Pain is an unpleasant sensory and emotional experience associated with actual or potential tissue damage, or described in terms of such damage (Loeser and Melzack, 1999; Raja et al., 2020). Meanwhile, opioids, owing to their potent analgesic properties, play a dominant role in the management of acute and chronic pain, particularly in perioperative settings (Corder et al., 2018). However, this therapeutic advantage is accompanied by significant clinical challenges: the global crisis of opioid misuse and addiction continues to escalate (Demsey et al., 2017), while side effects such as opioid tolerance and OIH(Mercadante et al., 2019) resulting from overuse have become major concerns in anesthesiology (Richards et al., 2020).

Mechanistic studies have revealed that the effects of opioids extend far beyond the direct modulation of μ-receptors. Animal experiments demonstrate that prolonged morphine exposure activates the pyruvate dehydrogenase kinase 4/phosphorylated pyruvate dehydrogenase axis in spinal astrocytes, leading to lactate overload. This, in turn, triggers neuronal sensitization via the astrocyte-neuron lactate shuttle (Ma et al., 2024). This glial-neuronal interaction provides a molecular-level explanation for paradoxical phenomena such as OIH, suggesting that the dual effects of opioids—analgesia and hyperalgesia—are rooted in complex cellular networks.

Clinical evidence further highlights the contradictory nature of opioid use. While morphine, fentanyl, and other opioids remain foundational for managing moderate to severe pain (e.g., cancer pain, postoperative pain), their dose-limiting side effects (e.g., respiratory depression, constipation) significantly constrain therapeutic benefits. A prospective observational study revealed that 54% of patients with preoperative opioid exposure continued opioid use 3 months post-surgery, with no significant correlation to surgical site pain (Kuck et al., 2023). This raises questions about the long-term necessity of opioids in chronic pain management.

Faced with this dual nature, clinical practice is caught in a dilemma: although opioid reduction strategies have shown initial progress, completely avoiding opioid use in perioperative settings remains technically challenging (Shanthanna and Joshi, 2024). Ongoing debates surrounding OFA efficacy (Mulier, 2019) and the lack of standardized guidelines underscore the urgent need for breakthroughs in this field. The core challenge lies in achieving adequate analgesia while mitigating the neurobiological cascades and long-term adverse outcomes induced by opioids.

#### 4.2.2 PONV

The cited references and keyword cluster analysis indicate that PONV is a critical research theme in the field of OFA. The mechanisms underlying PONV and its prevention strategies urgently need clarification. Its etiology is multifaceted: the primary risk stems from the combined use of intraoperative opioids and inhalational anesthetics (Binning et al., 2011; Apfel et al., 2012; Horn et al., 2014), while the inflammatory response triggered by surgical trauma further exacerbates this complication. PONV not only reduces patient comfort but, in rare cases, can also lead to complications such as dehydration, electrolyte imbalances, aspiration of gastric contents, esophageal rupture, suture dehiscence, and hemorrhage. This has driven clinical exploration of OFA as an alternative approach.

Current evidence suggests that the benefits of OFA in PONV prevention are procedure-specific. In upper limb surgeries on obese patients, OFA reduces the frequency of PONV episodes, decreases antiemetic use, shortens extubation time, and lowers pain scores (Ahmed Abdelghaffar et al., 2024). In thoracoscopic lung resections, OFA reduces the incidence of PONV from 31.7% to 15%, albeit with longer PACU stays (Feng et al., 2024). In thyroid/parathyroid surgeries, OFA lowers the 48-hour PONV incidence to 5% (compared to 24% in opioid users), reduces the need for rescue antiemetics, decreases post-extubation hypotension and desaturation rates, and is associated with higher patient satisfaction (Wang et al., 2024). In contrast, OFA fails to reduce PONV incidence in certain procedures (e.g., gynecologic laparoscopy (Massoth et al., 2021), video-assisted thoracoscopic surgery (Bao et al., 2024), cholecystectomy (Sinclair et al., 1999; Toleska et al., 2022; Chen H. Y. et al., 2023)), often accompanied by prolonged PACU stays or hemodynamic instability. This heterogeneity in efficacy underscores the need for individualized decision-making based on surgical type (e.g., high-risk procedures like laparoscopy and orthopedic shoulder surgeries) and patient characteristics (e.g., age, gender, smoking history). Meta-analyses confirm that OFA offers superior early PONV control in laparoscopic bariatric surgeries compared to traditional anesthesia, though the risk of bradycardia must be weighed (Ao et al., 2025).

Beyond anesthetic choices, PONV occurrence is influenced by multiple variables: a 10-year increase in age reduces the risk by 13%; males have one-third the risk of females; a 30-minute increase in anesthesia duration raises the risk by 59%; and general anesthesia increases the risk 11-fold compared to other methods. Notably, postoperative opioid use (e.g., patient controlled analgesia or epidural analgesia) also significantly elevates PONV incidence (Mao et al., 2025).

Although enhanced recovery after surgery protocols have integrated MMA strategies, PONV remains a critical bottleneck affecting postoperative outcomes. Emerging interventions, such as perioperative low-dose naloxone combined with opioids (Mao et al., 2025) and intraoperative acupuncture-assisted analgesia (Zhang et al., 2022), show promise in PONV prevention by blocking opioid receptors or reducing opioid use. Future research must focus on establishing procedure-specific stratification criteria for OFA indications, developing predictive models that integrate patient characteristics and real-time monitoring data, and exploring the synergistic mechanisms of non-opioid analgesia and antiemetic effects.

### 4.3 Research Frontiers

Through a comprehensive analysis of keyword clusters and keywords timeline view, identifying their inherent patterns and connections can greatly contribute to elucidating global trends and research frontiers in the field of OFA. Studies on evoflurane anesthesia, plane block, MMA, and OSA are currently at the frontiers of academic research, highlighting the necessity for further investment in this area.

#### 4.3.1 Sevoflurane anesthesia

As an inhaled anesthetic, sevoflurane has emerged as a cornerstone agent for maintaining anesthetic depth in OFA due to its rapid induction, smooth emergence, and hemodynamic stability (Kim et al., 2011). Its core mechanism lies in partially replacing the analgesic effects of opioids through inhibiting NMDA receptors (Brosnan and Thiesen, 2012) in the central nervous system and enhancing GABAergic neurotransmission (Philip et al., 2025). This unique pharmacological profile has propelled sevoflurane to the Frontier of OFA research in recent years.

Clinical applications have demonstrated validated synergism between sevoflurane and dexmedetomidine. In thoracic procedures such as video-assisted pulmonary lobectomy, OFA protocols combining sevoflurane with dexmedetomidine and esketamine achieved superior outcomes compared to traditional opioid-based regimens, notably reducing PONV incidence (Feng et al., 2024) while avoiding opioid-induced immune suppression. Of particular clinical significance is the dose-response relationship. 5% sevoflurane induction effectively reduces sufentanil-induced cough in pediatric patients by achieving bispectral index (BIS) values around 60 without significant hemodynamic fluctuations (Ji et al., 2025). Advanced administration techniques like BIS-guided titration have shown efficacy in optimizing clinical outcomes. Pediatric trials confirmed this approach decreases end-tidal sevoflurane concentrations by 15%–20% while maintaining intraoperative hemodynamic stability (heart rate, mean arterial pressure), consequently reducing postoperative delirium incidence (Frelich et al., 2024). Such precision dosing proves particularly advantageous in ambulatory surgeries requiring rapid recovery.

Nevertheless, sevoflurane-based OFA faces multiple challenges. First, its solitary application remains controversial in completely suppressing surgical stress responses. Studies on pediatric cardiac catheterization have shown that emergence agitation may occur after sevoflurane anesthesia, necessitating the use of non-opioid adjuncts such as dexmedetomidine to reduce its incidence (Hassan and Saleh, 2024). Second, pharmacological trade-offs require meticulous evaluation. While low-flow administration (<2 L/min) potentially reduces oxidative damage via suppressed disulfide bond formation (Kaşıkara et al., 2022), concurrent nephrotoxicity risks demand vigilant monitoring (Kharasch et al., 2005). Furthermore, fluoride ion accumulation during prolonged procedures (Gallego et al., 2015) and its oncological implications in cancer patients await validation through multicenter cohort studies.

More critically, emerging evidence elucidates dose-dependent neurocognitive impacts. Murine models demonstrate that low-concentration sevoflurane exposure exacerbates microglial pyroptosis (Lin et al., 2024) through hippocampal HDAC6-mediated HSP90/70-NLRP3 inflammasome activation, leading to spatial memory deficits and CA1 neuronal loss (Yamamoto et al., 2023).

In conclusion, while sevoflurane demonstrates substantial clinical advantages in OFA implementation, its application requires meticulous protocol customization based on patient profiles and surgical requirements. Future research should prioritize elucidating synergistic mechanisms with non-opioid adjuvants and developing real-time monitoring-guided personalized administration systems, thereby advancing standardized OFA protocols across diverse surgical disciplines.

#### 4.3.2 Plane block

The analysis of the timeline view of keywords shows that plane block is a key research Frontier in OFA. With the development of MMA and OFA (Jiao et al., 2022), the plane block technique has been substantially improved. The application of diverse plane block techniques during the perioperative period can serve as an alternative to traditional analgesia, reducing the reliance on opioids while facilitating the advancement of OFA. Transversus abdominis plane block can play an analgesic role in abdominal surgery (Ibrahim et al., 2022). Erector spinae plane block can be used in spinal surgery (Taşkaldıran, 2021; Ragupathy et al., 2022) and hepatectomy (Elshafie et al., 2022). Erector spinae plane block and paravertebral block can enhance postoperative rehabilitation following total hip replacement (Kolomachenko, 2022), and quadratus lumborum block is beneficial for postoperative analgesia and intraoperative opioid-sparing in lower abdominal or pelvic surgery (Kolomachenko, 2022). Specifically, it is a method of MMA, but it has significant potential for advancement and development, particularly in the context of OFA.

#### 4.3.3 MMA

Keywords co-occurrence and timeline view of keywords map analysis showed that MMA was a key research Frontier in OFA.

MMA is defined as a combination of narcotics and analgesics, although the different drugs or techniques work by different mechanisms, but are designed to provide additional or synergistic pain relief while minimizing the adverse effects associated with higher doses of opioids. MMA is defined as the combination of anesthetics and analgesics (Kehlet and Dahl, 1993), with different drugs or techniques working by different mechanisms, all aiming to achieve pain relief through additional or synergistic effects, while also minimizing the adverse effects associated with high doses of opioids (Shanthanna et al., 2021). Surgeons actively promote postoperative MMA by employing techniques such as local infiltration or insertion of surgical site catheters (Brown et al., 2018). MMA is delivered through the use of medications or anaesthetic techniques (Lersch et al., 2023), such as NSAIDs, dexmedetomidine, lidocaine, ketamine and propofol, and sevoflurane.

NSAIDs could exert their effects by reducing inflammation and providing analgesia. In terms of mechanisms, NSAIDs possess the capacity to inhibit the activity of cyclooxygenase isoforms 1 and 2 and disrupt the production of prostaglandins, thereby reducing the production of these inflammatory mediators and noxious substances (Vane, 1971).Studies have shown that the use of NSAIDs before surgery reduces postoperative pain and postoperative opioid consumption (Munro et al., 2018).

Dexmedetomidine is a potent and highly selective α2 adrenergic receptor agonist with sympatholytic, amnestic, and analgesic effects (Janssen et al., 2019; Afonso and Reis, 2012). It plays a key anti-neurotoxic role by activating inhibitory interneurons to enhance descending inhibition of nociceptive transmission and reduce arousal. Furthermore, dexmedetomidine has been shown to induce a distinctive form of “conscious sedation,” characterized by the absence of respiratory depression. The primary mechanism of ketamine involves the targeting of N-methyl-D-aspartate (NMDA) glutamate receptors on peripheral afferent nociceptive neurons at synapses in the dorsal horn of the spinal cord, and its primary clinical applications include the management of pain, the reduction of opioid requirements, and also exhibiting antidepressant properties (Sinner and Graf, 2008). Lidocaine, a commonly used local anesthetic, is often used as an adjunct to help control pain during and after surgical procedures. When used for nerve block or local anesthesia, it produces anti-neonociception by inhibiting the excitation of nerve endings or by blocking the conduction of action potentials in peripheral nerves. The intraoperative administration of dexmedetomidine has been demonstrated to effectively reduce the incidence of postoperative delirium in patients undergoing elective noncardiac surgery. The use of dexmedetomidine, ketamine, and lidocaine during surgery is also capable of reducing the consumption of postoperative opioids and the incidence of PONV. Lidocaine and ketamine can also help reduce postoperative pulmonary complications (Carron et al., 2024) and postoperative chronic pain (Doleman et al., 2023).

Propofol and sevoflurane are the main sedatives and hypnotics utilized in contemporary anesthesia protocols, which mainly inhibit the synapses of interneurons through γ-aminobutyric acid subtype A (GABAA) receptors (Bowery et al., 1987). These drugs reduce the perception of nociceptive stimuli by rendering the patient unconscious, although they do not act directly on nociceptive pathways.

#### 4.3.4 OSA

Analysis of the Keywords Timeline View reveals that OSA represents a significant research Frontier. The fundamental principle of OSA involves achieving perioperative analgesia through the minimal intraoperative use of opioids combined with non-opioid adjuvants (Lauretta et al., 2025). Studies have demonstrated that in laparoscopic gynecologic surgeries, OSA significantly reduces PONV, pain scores, and rescue analgesic requirements in the post-anesthesia care unit (PACU), compared to traditional opioid-based anesthesia, without increasing hemodynamic instability (Nam et al., 2024). These findings are corroborated by additional research (Anyaehie et al., 2022; Lauretta et al., 2025).

The emergence of OSA and OFA reflects a paradigm shift toward MMA and reduced opioid reliance in perioperative care. Recent RCTs have directly compared the clinical efficacy of these two strategies, providing critical evidence for anesthesia practice. For instance, a triple-blind RCT by Chassery et al. (Chassery et al., 2024) compared OFA (dexmedetomidine-based) with OSA (sufentanil-based) in outpatient total hip arthroplasty. Results showed no significant differences in 24-hour postoperative oral morphine equivalents consumption, pain scores, or early recovery metrics between groups. This suggests that intraoperative opioid avoidance (OFA) offers no additional benefit over OSA when optimized MMA strategies (e.g., NSAIDs, local anesthetic infiltration) are employed, potentially due to its limited impact on postoperative opioid demand (Shanthanna et al., 2024). Further analysis revealed comparable rates of opioid-related adverse effects (e.g., PONV) between OSA and OFA, likely attributable to low intraoperative opioid doses in both groups (Chassery et al., 2024). Notably, the OFA group exhibited significantly lower intraoperative bradycardia incidence linked to dexmedetomidine, implying that non-opioid adjuvants (e.g., α2-agonists) may enhance recovery metrics by modulating sympathetic tone (Liu et al., 2019; Chassery et al., 2024).

Similarly, an RCT in obese patients undergoing laparoscopic sleeve gastrectomy found no statistical differences in 48-hour morphine consumption, pain scores, or antiemetic requirements between OFA and OSA (Barakat et al., 2025). However, the OFA group required more antihypertensive interventions to maintain hemodynamic stability. This paradoxical outcome highlights potential challenges in specific populations (e.g., obesity/metabolic syndrome), possibly related to dexmedetomidine- or ketamine-induced sympatholytic effects (Wang et al., 2019; Shanthanna et al., 2024).

From an evidence-based perspective, Shanthanna et al. (2024) emphasize that while OFA reduces PONV in minimally invasive abdominal surgeries, its improvements in opioid consumption and pain scores lack clinical significance despite statistical differences. This implies that OFA’s benefits may be restricted to select outcomes (e.g., high PONV-risk patients), whereas OSA’s “minimum effective dose” strategy balances analgesia and safety, remaining a more universally applicable approach.

In addition, both studies acknowledge limitations in statistical power. For example, Chassery et al. (Hublet et al., 2022)’s post-hoc sample size calculation indicated a requirement of 254 patients to detect significant OME differences, far exceeding their 40-patient cohorts, thereby undermining reliability.

In conclusion, current evidence suggests that OSA and OFA achieve comparable opioid minimization within MMA frameworks, with no clear superiority of OFA over OSA. Clinical decision-making should integrate surgical context, patient comorbidities (e.g., obesity, obstructive sleep apnea), and agent-specific risks (e.g., hemodynamic fluctuations). Future research priorities include large-scale multicenter RCTs and exploration of phenotype- or genotype-guided personalized analgesia to refine opioid stewardship.

## 5 Conclusion

Based on bibliometrics and visual analysis, this study comprehensively, scientifically and systematically analyzes the current research status and frontiers of OFA. OFA is undergoing rapid development in the field of anesthesiology, attracting increasing attention and thus promoting further advancement. According to our study, current trends and research topics are focused on pain, opioid consumption, and PONV. The prospective frontiers of research will encompass domains pertaining to sevoflurane anesthesia, plane block, MMA, and OSA. The findings of this study provide a theoretical reference and guidance for the future development of OFA in clinical anesthesia.

## 6 Limitations

Inevitably, this study has some limitations. First, only the WoSCC database was used as a data source. Data from other large databases such as PubMed, Scopus, and Embase were excluded. Second, our study focused only on articles and review articles, and other types of publications were excluded, which may have led to the missingness of relevant studies. In addition, due to the relatively low citation rates, some high-quality studies published recently may have been underestimated. Finally, bibliometric analyses are unable to analyze key factors such as the design ideas of the articles as well as the experimental methods, which is a limitation in terms of research quality.
